# High-resolution late gadolinium enhancement imaging with compressed sensing: a single-center clinical study

**DOI:** 10.1186/1532-429X-18-S1-O56

**Published:** 2016-01-27

**Authors:** Mehmet Akcakaya, Tamer Basha, Connie Tsao, Sophie Berg, Kraig V Kissinger, Beth Goddu, Warren J Manning, Reza Nezafat

**Affiliations:** 1 grid.17635.360000000419368657Electrical and Computer Engineering, University of Minnesota, Minneapolis, MN USA; 2 grid.17635.360000000419368657Center for Magnetic Resonance Research, University of Minnesota, Minneapolis, MN USA; 3 grid.239395.70000000090118547Medicine (Cardiovascular Division), Beth Israel Deaconess Medical Center, Boston, MA USA; 4 grid.7776.10000000406399286Biomedical Engineering, Cairo University, Giza, Egypt; 5 grid.239395.70000000090118547Radiology, Beth Israel Deaconess Medical Center, Boston, MA USA

## Background

Late gadolinium enhancement (LGE) MRI is the clinical standard for imaging of scar in the left ventricle (LV). It has also been employed for assessing RF ablations in the left atrium (LA). LV LGE imaging is typically performed in 2D. When 3D is utilized, spatial resolution is limited due to prolonged scan time. We have shown that an accelerated imaging technique called LOST with random undersampling can be used to achieve isotropic resolution in 3D LGE. To benefit from this novel technique and the resolution gains, reconstruction needs to be automated and integrated into clinical workflow. In this study, we sought to utilize random undersampling and LOST reconstruction, with a novel software tool that enables clinically feasible integration, in order to enable LGE MRI with high isotropic spatial resolution in the clinical setting in a large cohort of patients.

## Methods

### Patient Study

In a prospective study, 270 patients (181 men; 54.9 ± 14.1 yrs) were recruited. 190 were referred for LV LGE, and 80 for LA LGE. LGE were acquired 10-to-20 minutes after bolus infusion of 0.1-to-0.2 mmol/kg of Gd-BOPTA or Gd-DTPA. Imaging was performed at 1.5T with an ECG-triggered navigator-gated IR-GRE sequence. The isotropic resolution varied from 1 × 1 × 1 mm^3^ to 1.5 × 1.5 × 1.5 mm^3^. 3D random undersampling was used, with acceleration rates of 3 (133 patients) or 5 (137 patients), corresponding to acquisition durations of 4:00 or 2:30 minutes (70 bpm, 100% navigator efficiency).

### Reconstruction

**Figure 1:**
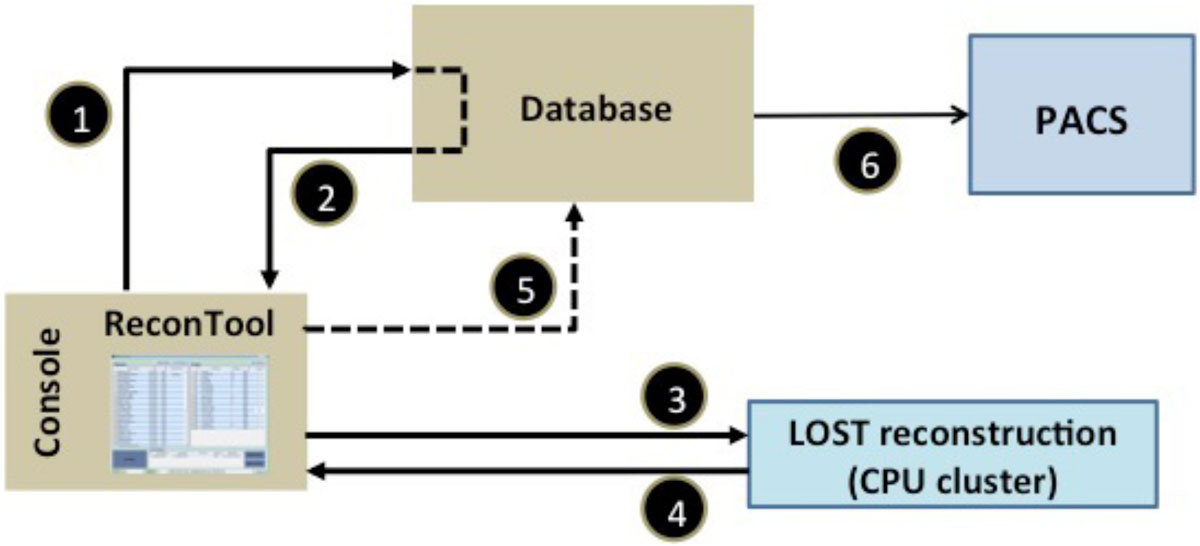
**Integration of the automated reconstruction framework into the clinical workflow**: (1) The scanner database is inquired for patients, scans, and raw data, (2) The database replies with the information, (3) Raw data is packed and sent to the CPU cluster for LOST reconstruction, (4) Progress & results are updated upon request from the operator, (5) Reconstructed images are pushed to the scanner database, (6) Reconstructed images are sent to the hospital PACS.

Randomly undersampled acquisitions were reconstructed offline using B_1_-weighted LOST algorithm as in. The automated reconstruction framework (Fig. 1) was implemented based on. After an LGE scan the operator used the framework to initiate LOST reconstruction on a remote CPU cluster. The reconstruction progress was updated via communication with the cluster, while the operator continued scanning. After finishing, results were automatically retrieved, sent to the scanner database and stored on hospital PACS.

### Analysis

Subjective image quality assessment was performed by an experienced blinded cardiologist. Presence of LGE was assessed as present with confidence/absent with confidence/unconfident to decide. Datasets were also assessed using a 4-point system: 1 (poor, LGE interpretable in < 50% of LV myocardium/LA), 2 (fair, LGE interpretable in 50-75% of LV/LA), 3 (good, LGE interpretable in 75-90% of LV/LA), 4 (excellent, LGE interpretable in >90% of LV/LA).

## Results

**Figure 2:**
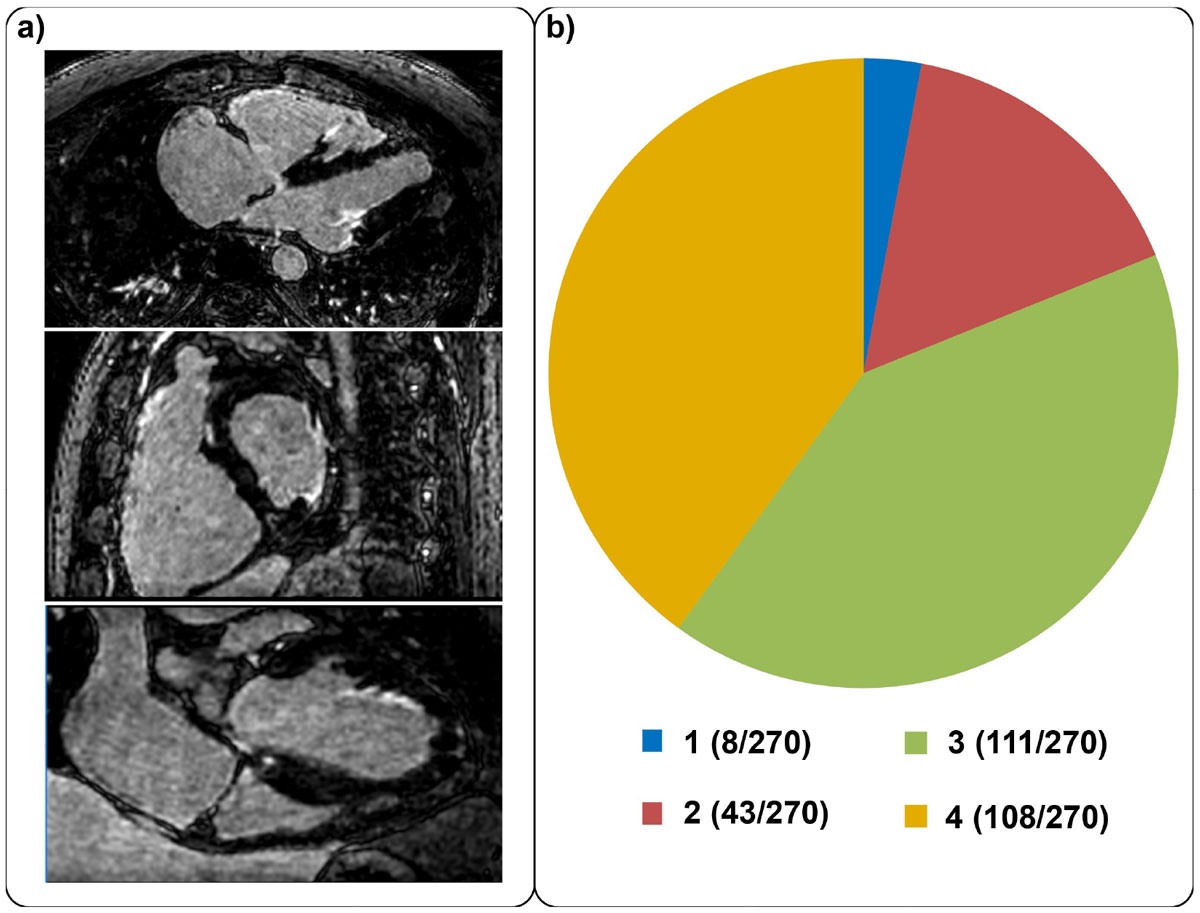
**a) Isotropic 3D LGE image acquired at 1 × 1 × 1 mm**^**3**^ **resolution, viewed in axial, sagittal and coronal views.** The enhancement is clearly visible in all orientations. **b)** The summary of the subjective image quality readings (1-poor, 2-fair, 3-good, 4-excellent), showing a large number of studies (229/270) were scored favorably as good or excellent.

LGE imaging was completed in all cases. Enhancement was visually present in 49 patients, absent in 214 patients, and the reader was unconfident in 7 cases. Fig. 2a shows an example LGE image in axial, sagittal and coronal views. Fig. 2b shows the results of the qualitative assessment, where >84% of the cases were scored good or excellent.

## Conclusions

The proposed acquisition and automated reconstruction framework enables the clinical use of accelerated high isotropic resolution LGE imaging in a large number of patients.

